# Reward Value Determines Memory Consolidation in Parasitic Wasps

**DOI:** 10.1371/journal.pone.0039615

**Published:** 2012-08-22

**Authors:** H. Marjolein Kruidhof, Foteini G. Pashalidou, Nina E. Fatouros, Ilich A. Figueroa, Louise E. M. Vet, Hans M. Smid, Martinus E. Huigens

**Affiliations:** 1 Department of Terrestrial Ecology, Netherlands Institute of Ecology (NIOO-KNAW), Wageningen, The Netherlands; 2 Laboratory of Entomology, Wageningen University, Wageningen, The Netherlands; 3 Fundación PROINPA, Cochabamba, Bolivia; Alexander Flemming Biomedical Sciences Research Center, Greece

## Abstract

Animals can store learned information in their brains through a series of distinct memory forms. Short-lasting memory forms can be followed by longer-lasting, consolidated memory forms. However, the factors determining variation in memory consolidation encountered in nature have thus far not been fully elucidated. Here, we show that two parasitic wasp species belonging to different families, *Cotesia glomerata* (Hymenoptera: Braconidae) and *Trichogramma evanescens* (Hymenoptera; Trichogrammatidae), similarly adjust the memory form they consolidate to a fitness-determining reward: egg-laying into a host-insect that serves as food for their offspring. Protein synthesis-dependent long-term memory (LTM) was consolidated after single-trial conditioning with a high-value host. However, single-trial conditioning with a low-value host induced consolidation of a shorter-lasting memory form. For *Cotesia glomerata*, we subsequently identified this shorter-lasting memory form as anesthesia-resistant memory (ARM) because it was not sensitive to protein synthesis inhibitors or anesthesia. Associative conditioning using a single reward of different value thus induced a physiologically different mechanism of memory formation in this species. We conclude that the memory form that is consolidated does not only change in response to relatively large differences in conditioning, such as the number and type of conditioning trials, but is also sensitive to more subtle differences, such as reward value. Reward-dependent consolidation of exclusive ARM or LTM provides excellent opportunities for within-species comparison of mechanisms underlying memory consolidation.

## Introduction

Rewards (e.g. food, hosts) of different value offered during associative conditioning are known to induce changes in the conditioned response, such as floral odor preference in honey- and bumblebees [1], [2], the duration of a feeding response to odors in parasitic wasps [3], win-shift tendency in birds [4], place preference in rats [5] and cache recovery preference in food-storing birds [6], as well as changes in the duration of memory for, e.g., odors in fruit flies [7]. The value of a rewarding reinforcer can vary in relation to its relative contribution to an animal's fitness and the reliability of the association between the learned information and the reward. We hypothesized that rewards of the same type but of different value may also induce consolidation of a different memory form. Consolidation of long-term memory may not be optimal when the reward value is relatively low, because it is energetically more costly than consolidation of shorter-lasting memory forms [8] and because instant consolidation of long-term memory would not be advantageous if the risk of making irrelevant associations is high [9].

In insects it has been shown that initial, labile anesthesia-sensitive memory (ASM) can be consolidated into ARM and/or more stable, protein synthesis-dependent LTM [10]–[14]. In contrast to ASM, the formation of both ARM and LTM is insensitive to retrograde amnesia applied immediately after conditioning [15]. Hence, both ARM and LTM are regarded as forms of “consolidated memory”. LTM differs from ARM in that only the consolidation of LTM is dependent on ‘de novo’ protein synthesis, which can be blocked by protein-synthesis inhibitors, and involves structural changes in the brain [10]. Molecular mechanisms of memory formation are highly conserved throughout the animal kingdom [16], although the nature of the disruptive treatments that are used to distinguish between memory forms may slightly differ. Also in vertebrates protein-synthesis inhibitors, such as anisomycin, are used to prevent the formation of LTM [17]. However, while cold-shock is often used in invertebrates to induce retrograde amnesia [18], [19], electroconvulsive shocks or CO_2_ are more widely used in homeothermic animals [20], [21]. Which memory form is consolidated is known to depend on the number of conditioning trials (single versus multiple trials spaced in time) and the type of conditioning (aversive conditioning using a punishing reinforcer versus appetitive conditioning using a rewarding reinforcer) [10]–[12]. Whether more subtle differences in the reinforcer can also affect memory consolidation is currently still unknown.

Parasitic wasps (parasitoids) are ideal model organisms for studying the effect of reward value on memory consolidation in an ecologically relevant context. These insects optimize searching efficiency for their inconspicuous hosts by learning to associate odor cues with host presence [13], a learning task that is intricately linked to fitness. The reward in this context is egg-laying into the host (Movies S1, S2). The odor cues that are learned differ between parasitoids of insect larvae and insect eggs: egg parasitoids can exploit pheromones emitted by adult mated female hosts and hitch-hike with them to egg-laying sites [22]–[24] (Movie S3), whereas parasitoids of insect larvae can respond to plant odors induced by feeding host larvae [25].

**Figure 1:**
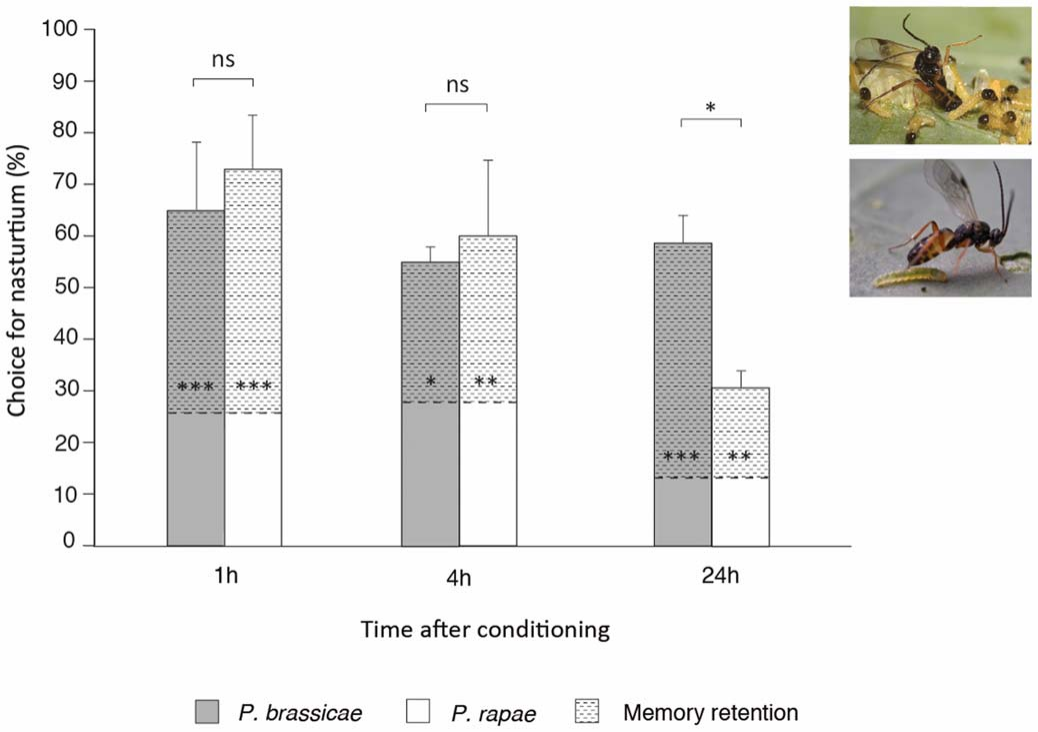
*Cotesia glomerata* memory retention after single-trial conditioning with a low- or high-value reward. Mean percent choice (+ SE) of *Cotesia glomerata* for nasturtium (learned odor) at 1 h, 4 h and 24 h after single-trial conditioning with *Pieris brassicae* (high-value reward; dark grey bars) or *P. rapae* (low-value reward; white bars). *Cotesia glomerata* was conditioned by allowing each wasp to lay eggs into one newly-emerged *Pieris* caterpillar that was placed on either a host-damaged leaf of a nasturtium plant (*Tropaeolum majus*) or a Brussels sprouts plant (*Brassica oleracea* var. gemmifera) [41]. Wasps were tested in a two-choice wind tunnel set-up [42] in which they could fly towards, and land on, a nasturtium or a Brussels sprouts plant. Brussels sprouts odor is relatively more attractive to inexperienced wasps than nasturtium odor [35] and this preference cannot be further increased through conditioning with Brussels sprouts [15]. We used the percentage choice for nasturtium of wasps conditioned on Brussels sprouts as a reference (indicated by the dotted lines inside the bars), as these wasps have a higher flight response towards the plants than inexperienced wasps [9], [36], [41]. Mean reference values (± SE) are 25.7 (±14.12) for 1 h, 27.5 (±9.46) for 4 h and 13.3 (±4.64) for 24 h. Memory was defined as present when the percentage choice for nasturtium of wasps conditioned with the nasturtium odor was significantly higher than the reference (indicated by asterisks inside the bars), with a larger difference representing a higher memory retention level. Memory retention is indicated by the dashed pattern inside the bars. For each treatment we tested 10–15 *C. glomerata* wasps on at least 4 different days (n>40). Each wasp was tested only once. * = *P*<0.05; ** = *P*<0.01; *** = *P*<0.001; ns = not significant (GLM using the variable ‘memory retention’ for comparisons between bars (Table 2) and GLM with logit-link function using the variable ‘percentage choice for nasturtium’ for comparisons within bars (Table 1)). Photos represent a *C. glomerata* wasp parasitizing a *P. brassicae* caterpillar within a cluster (above) or a single *P. rapae* caterpillar (below). Photos courtesy of Hans M. Smid (www.bugsinthepicture.com).

**Figure 2:**
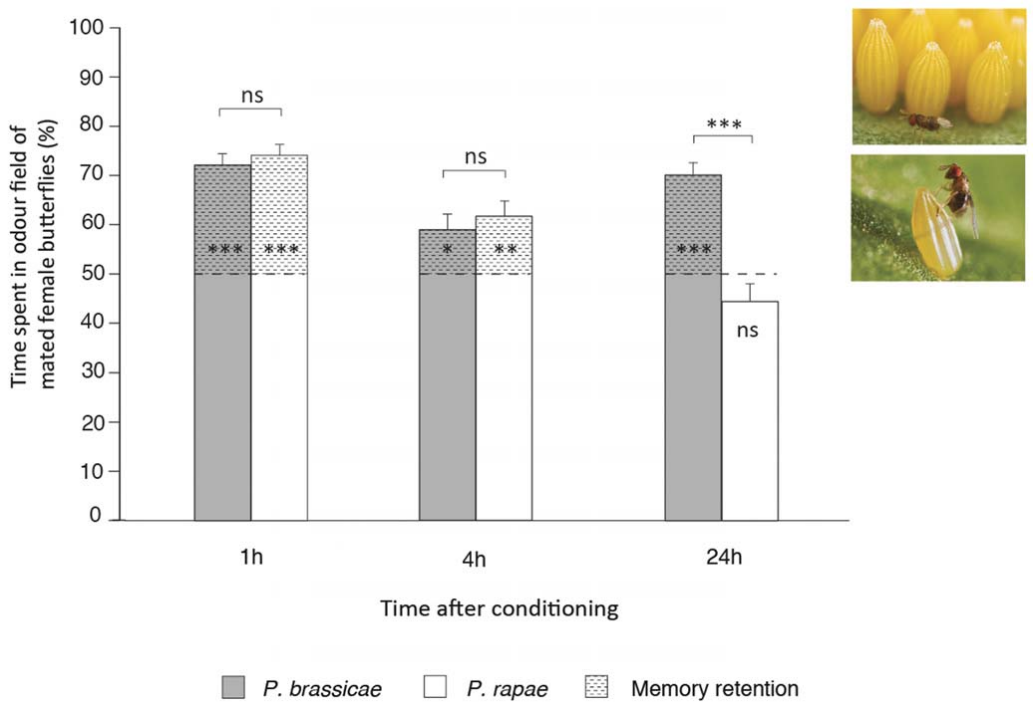
*Trichogramma evanescens* memory retention after single-trial conditioning with a low- or high-value reward. Mean percent time (+ SE) spent in odor field of mated female butterflies (learned odor) of *Trichogramma evanescens* at 1 h, 4 h and 24 h after single-trial conditioning with *Pieris brassicae* (high-value reward; dark grey bars) or *P. rapae* (low-value reward; white bars). *Trichogramma evanescens* was conditioned by allowing each wasp to approach and mount a recently mated (and thus egg-laying) female butterfly, emitting an anti-aphrodisiac pheromone, followed by the reward: parasitizing one freshly laid single *P. rapae* egg or one *P. brassicae* egg within an egg cluster [23], [24]. Wasps were tested in a 2-chamber olfactometer [23], [24] with two mated female butterflies in one compartment and two males in the other. As these two odors are equally attractive to inexperienced wasps [23], [24], we used 50% of time spent in the odor field of mated female butterflies as a reference (indicated by the dotted line inside or above the bars). Memory was defined as present when the percentage of time spent in the odor field of mated female butterflies was significantly higher than the reference (indicated by asterisks inside the bars), with a larger difference representing a higher memory retention level. Memory retention is indicated by the dashed pattern inside the bars. For each treatment we tested a total of 40 *T. evanescens* wasps. Each wasp was tested only once. * = *P*<0.05; ** = *P*<0.01; *** = *P*<0.001; ns = not significant (GLM for comparisons between bars (Table 3) and WMPSRT for comparisons within bars). Photos represent a *T. evanescens* wasp parasitizing a *P. brassicae* egg within a cluster (above) or a single *P. rapae* egg (below). Photos courtesy of Nina E. Fatouros (www.bugsinthepicture.com).

In this study, we investigated the effect of host-species (i.e. reward) on memory consolidation in two parasitic wasp species belonging to different families, *Cotesia glomerata* (Hymenoptera: Braconidae) and *Trichogramma evanescens* (Hymenoptera: Trichogrammatidae; Fig. 1, 2). These wasps parasitize the larvae and eggs, respectively, of two closely related cabbage white butterflies; *Pieris brassicae*, which lays egg clusters [26], and *P. rapae*, which deposits single eggs [27]. Both *C. glomerata* and *T. evanescens* wasps readily consolidate LTM after single-trial conditioning with *P. brassicae* [9], [23]. *Pieris brassicae* hosts offer a higher contribution to the maternal fitness of the two parasitic wasp species than *P. rapae* hosts, both for quantitative and qualitative reasons. First of all, a female parasitoid needs to find relatively few *P. brassicae* clusters to lay all her eggs, whereas she needs to locate a large number of single *P. rapae* hosts to do the same. Furthermore it has been shown for both parasitic species that one *P. brassicae* host (caterpillar or egg) gives rise to more and larger parasitoid offspring than one *P. rapae* host [28], [29]. We therefore hypothesized that single-trial conditioning with *P. rapae* will not result in LTM consolidation, but that instead only the shorter-lasting memory form ARM is consolidated.

## Results

### Memory Retention

We first tested for the presence of memory at different times after conditioning with either *P. brassicae* or *P. rapae* by comparing the percentage choice for nasturtium between wasps conditioned with the odor of a nasturtium plant and the reference group that was conditioned with the odor of a Brussels sprouts plant (*C. glomerata*; comparisons within bars in Fig. 1) or by assessing whether the difference in percentage of time spent in the odor field of mated female butterflies differs from 50% (*T. evanescens;* comparisons within bars in Fig. 2). Next, we tested whether the memory retention (MR) level changes with time after conditioning and whether this is dependent on the host-species used for conditioning (comparisons between bars in Fig. 1, 2). We found memory in *C. glomerata* following single-trial conditioning with either *P. brassicae* or *P. rapae* to be present at all times tested (Table 1; comparisons within bars in Fig. 1) However, we found a significant effect on MR level of the interaction between host-species and time after conditioning (F_2,22_ = 5.70, *P = *0.010, Table 2; comparisons between bars in Fig. 1). Single-trial conditioning with the high-value host *P. brassicae* resulted in a significantly higher MR level than single-trial conditioning with the lower-value host *P. rapae* at 24 h after conditioning (Tukey-HSD, *P* = 0.016), while MR levels were comparable at 1 h (*P* = 0.960) and 4 h (*P* = 0.995) after conditioning. A similar difference in MR level was found for *T. evanescens* (interaction between host-species and time after conditioning (F_2,234_ = 15.29, *P*<0.001, Table 3; comparisons between bars in Fig. 2)). Also in *T. evanescens* was 24 h MR level significantly higher after single-trial conditioning with *P. brassicae* than after than single-trial conditioning with *P. rapae* (Tukey-HSD, *P*<0.001), whereas 1 h and 4 h MR levels were similar (*P* = 0.993 and *P* = 0.998, respectively) for both host-species. Memory was even completely absent in *T. evanescens* at 24 h after single-trial conditioning with *P. rapae* (percentage of time spent in the odor field of mated female butterflies = 44.3±3.54; Wilcoxon's matched-pairs signed-ranks test (WMPSRT), *P* = 0.116; comparison within 24 h bar in Fig. 2). These results indicate that both wasp species have longer-lasting memory after single-trial conditioning with *P. brassicae* than after single-trial conditioning with *P. rapae*.

**Table 1:** *Cotesia glomerata*; time series.

Time after conditioning	Treatment		
1 h	Conditioning treatment	X^2^_2 = _16.25	*P*<0.001
	• *P. rapae* on nasturtium		
	• *P. brassicae* on nasturtium		
	• *P. brassicae* on Brussels sprouts		
	Block (experimental day)*	X^2^_3 = _11.30	*P*<0.001
4 h	Conditioning treatment	X^2^_2 = _4.17	*P* = 0.016
	• *P. rapae* on nasturtium		
	• *P. brassicae* on nasturtium		
	• *P. brassicae* on Brussels sprouts		
	Block (experimental day)*	X^2^_3 = _3.00	*P* = 0.030
24 h	Host-species	X^2^_1 = _2.41	*P* = 0.121
	• *P. rapae*		
	• *P. brassicae*		
	Plant species used for conditioning	X^2^_1 = _43.79	*P*<0.001
	• nasturtium		
	• Brussels sprouts		
	Host-species * plant species used for conditioning	X^2^_1 = _6.49	*P* = 0.011
	Block (experimental day)*	X^2^_5 = _1.35	*P* = 0.238

* None of the interaction effects between block and treatment factors were significant (α = 0.05).

GLM with binomial distribution of error variance and a logit-link function. Response variable: Percentage choice for nasturtium (Fig. 1).

**Table 2:** *Cotesia glomerata*; time series.

Treatment		
Model	F_5,22 = _3.77	*P* = 0.013
Host-species	F_1,22 = _1.01	*P* = 0.325
• *P. rapae*		
• *P. brassicae*		
Time after conditioning	F_2,22 = _2.32	*P* = 0.122
• 1 h		
• 4 h		
• 24 h		
Host-species * time after conditioning	F_2,22 = _5.70	*P* = 0.010

GLM. Response variable: memory retention (results section).

**Table 3:** *Trichogramma evanescens*; time series.

Treatment		
Model	F_5,234 = _14.09	*P*<0.001
Host-species	F_1,234 = _9.27	*P* = 0.003
• *P. rapae*		
• *P. brassicae*		
Time after conditioning	F_2,234 = _15.31	*P*<0.001
• 1 h		
• 4 h		
• 24 h		
Host-species * time after conditioning	F_2,234 = _15.29	*P*<0.001

GLM. Response variable: Percentage of time spent in the odor field of mated female butterflies (Fig. 2). Data were arcsine square-root transformed to meet Levene's test for homogeneity of variance of treatment groups.

Subsequently we showed that both *C. glomerata* and *T. evanescens* are capable of forming 24 h-lasting memory with the lower-value reward *P. rapae* by comparing the choice behavior of wasps that received multiple conditioning trials spaced in time with *P. rapae* to the choice behavior of wasps that received a single conditioning trial with this host-species (X^2^_1_ = 721.95, *P*<0.001, Table 4). *Cotesia glomerata* wasps that received three spaced conditioning trials with *P. rapae* had significantly higher 24 h memory (percentage choice for nasturtium = 79.0±4.20) than wasps that received a single conditioning trial with this host-species (percentage choice for nasturtium = 27.5±4.78), and *T. evanescens* wasps that were given two spaced conditioning trials with *P. rapae* formed memory that lasted at least 24 h (percentage of time spent in the odor field of mated female butterflies = 58.3±3.88; WMPSRT, *P* = 0.013).

**Table 4:** *Cotesia glomerata*; spaced conditioning.

Treatment		
Conditioning	X^2^_1 = _721.95	*P*<0.001
• Single conditioning trial with *P. rapae*		
• Three conditioning trials with *P. rapae* spaced by 10 min intervals		
Block (experimental day)*	X^2^_3 = _73.16	*P*<0.001

* The interaction effect between block and conditioning treatment was not significant (α = 0.05).

GLM with binomial distribution of error variance and a logit-link function. Response variable: Percentage choice for nasturtium (results section).

### Memory Forms

**Figure 3:**
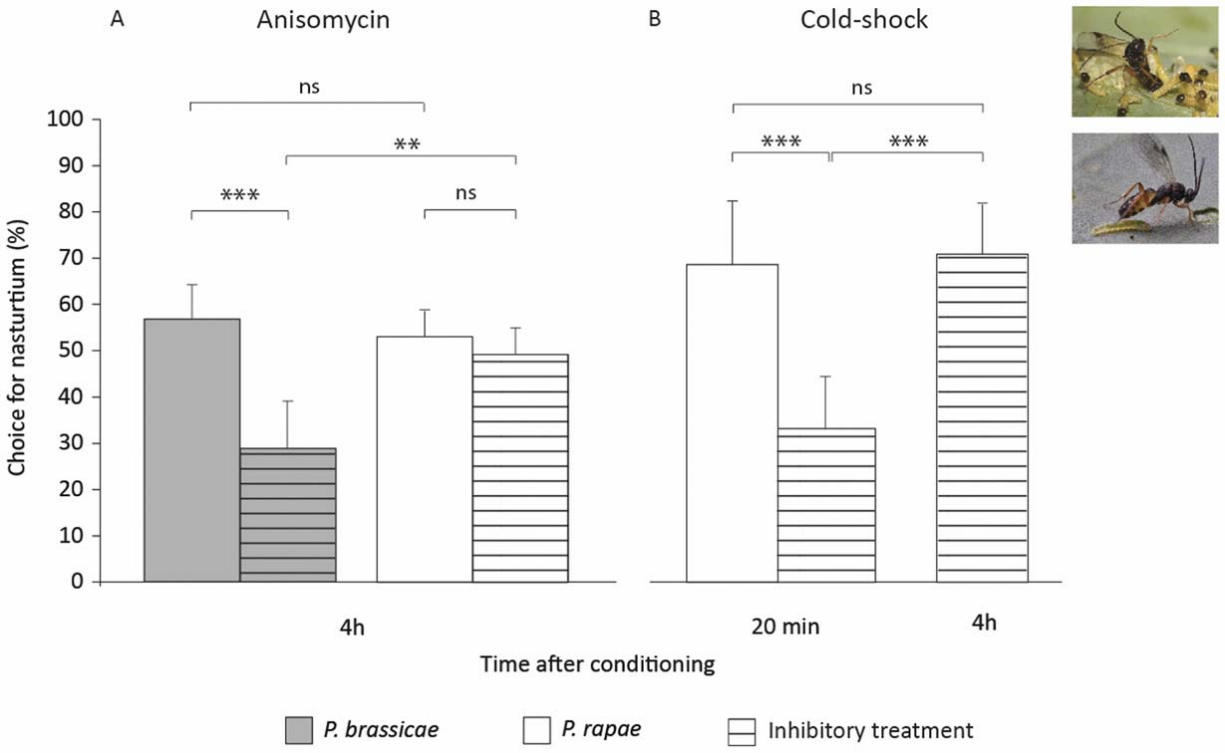
*Cotesia glomerata* memory dissection using inhibitory treatments. Effect of the LTM consolidation-inhibitor anisomycin (**A**) or an ASM-inhibiting cold-shock treatment (**B**) on the percentage choice for the learned odor in *Cotesia glomerata* at 20 min and/or 4 h after single-trial conditioning with *Pieris brassicae* (high-value reward; dark grey bars) or *P. rapae* (low-value reward; white bars) on a nasturtium plant. The inhibitory treatments are indicated by horizontal stripes. Wasps were tested in a two-choice wind tunnel set-up in which they could fly towards, and land on, a nasturtium or a Brussels sprouts plant. Brussels sprouts emits an odor that is relatively more attractive to inexperienced wasps than nasturtium odor [35]. For each treatment 10–15 wasps were tested on at least 4 different days (n>40). Each wasp was tested only once. * = *P*<0.05; ** = *P*<0.01; *** = *P*<0.001; ns = not significant (GLM with logit-link function, Table 5).

We further investigated for *C. glomerata* which memory form was consolidated after single-trial conditioning with *P. rapae*. We used the protein synthesis-inhibitor anisomycin to determine whether *P. rapae*-induced 4 h memory was protein synthesis-dependent. Since 4 h memory in *C. glomerata* following single-trial conditioning with *P. brassicae* is based on protein synthesis [15], we used wasps conditioned with *P. brassicae* to verify whether the anisomycin treatment was effective. We found a significant interaction between host-species and anisomycin treatment (X^2^_1_ = 5.91, *P* = 0.015, Table 5, Fig. 3A). As expected, anisomycin caused a significant reduction in the percentage choice for nasturtium of wasps conditioned with *P. brassicae* on this plant species (*P*<0.001). However, it did not affect the percentage choice for nasturtium of wasps conditioned with *P. rapae* on this plant (*P* = 0.592). This shows that in *C. glomerata* 4 h memory following a single-trial conditioning with *P. rapae* is not LTM.

**Table 5:** *Cotesia glomerata*; disruptive treatments.

Disruptive treatment	Treatment		
Anisomycin	Host-species	X^2^_1 = _2.85	*P* = 0.092
	• *P. rapae*		
	• *P. brassicae*		
	Treatment	X^2^_1 = _10.23	*P* = 0.001
	• Anisomycin		
	• Control		
	Host-species * treatment	X^2^_1 = _5.91	*P* = 0.015
	Block (experimental day)*	X^2^_3 = _3.17	*P* = 0.023
Cold-shock	Treatment	X^2^_2 = _8.74	*P*<0.001
	• *P. rapae* + cold-shock; test after 20 min		
	• *P. rapae* + cold-shock; test after 4 h		
	• *P. rapae* positive control; test after 20 min		
	Block (experimental day)*	X^2^_3 = _5.01	*P* = 0.002

* None of the interaction effects between block and treatment factors were significant (α = 0.05).

GLM with binomial distribution of error variance and a logit-link function. Response variable: Percentage choice for nasturtium (Fig. 3).

Subsequently, we investigated whether *P. rapae*-induced 4 h memory in *C. glomerata* is anesthesia-sensitive (ASM) or anesthesia-resistant (ARM). Cooling wasps on ice for 2 min immediately following conditioning on nasturtium disrupts ASM without affecting ARM or LTM consolidation [15]. Such treatment, therefore, results in a temporal amnesia (around 20 min after single-trial conditioning) until consolidated memory is present after 2–3 h [15]. We therefore compared the wasps' choice between nasturtium and Brussels sprouts at 20 min and 4 h after the cold-shock treatment, using different groups of wasps for each time point. Cold-shock applied 10 min before conditioning should not induce amnesia [30] and was used as a positive control for 20 min memory. We found a significant effect of cold-shock treatment (X^2^_2_ = 8.74, *P*<0.001, Table 5, Fig. 3B). As expected, the percentage choice for nasturtium of cold-shock treated wasps at 20 min after conditioning was reduced compared to the positive control treatment (*P*<0.001). However, the percentage choice for nasturtium of cold-shock treated wasps at 4 h after conditioning was not reduced (*P* = 0.812). This indicates that in *C. glomerata* 4 h memory following single-trial conditioning with *P. rapae* must be ARM.

## Discussion

Our results clearly show that two parasitic wasp species that belong to different hymenopteran families adjust their memory for odors in a similar manner to different-value rewards, represented by the same two host-species. This similarity in memory adjustment, regardless of the fact that the two species i) lay their eggs in different host life stages, i.e. in the host egg or caterpillar, ii) use different host (induced) odors, and iii) require different conditioning and memory testing procedures, indicates that reward value-dependent memory formation is not specific for a single species or a certain group of closely related parasitic wasps, but is likely to be a more general phenomenon. In both species, egg-laying in *P. brassicae* resulted in LTM consolidation, whereas egg-laying in *P. rapae* resulted in a shorter-lasting memory, identified as ARM in *C. glomerata*. *Pieris brassicae* offers a higher contribution to the maternal fitness of the two parasitic wasp species than *P. rapae*. A female parasitoid needs to find relatively few clusters of *P. brassicae* to lay all her eggs, whereas she needs to locate a large number of single *P. rapae* hosts to do so. *Trichogramma evanescens*, which immediately stops its hosts' development after egg-laying (i.e. is an idiobiont), can parasitize a wide range of host-species in addition to the two cabbage white species [31]. It assesses reward value by “measuring” egg- and cluster size while walking over the egg (cluster) [32] (Movie S2). As a single *P. brassicae* egg is larger than a *P. rapae* egg, a *P. brassicae* egg allows for the deposition of more eggs (3.4±0.11 (n = 91) and 2.9±0.07 (n = 45), resp.; Mann-Whitney U test, *P*<0.001) and/or the development of larger offspring [29]. For a koinobiont parasitoid like *Cotesia glomerata*, whose host lives until the developing wasps reach maturity (Movie S1), host size is not indicative of host quality [28]. However, *C. glomerata* has consistently higher developmental rate and larger clutch- and adult size when developing from a *P. brassicae* caterpillar than when developing from a *P. rapae* caterpillar [28]. As this wasp species primarily specializes on cabbage whites [33], it has likely evolved to value *P. brassicae* as a greater reward than *P. rapae*.

Moreover, *P. brassicae* constitutes a relatively high value host for *C. glomerata* in terms of the reliability of the association between the learned plant odors and the reward, because this butterfly mostly oviposits its egg clusters in clumps of vegetation of the same species [26]. This increases the chance that *C. glomerata* will find another host cluster on a nearby plant of the same species. In contrast, *P. rapae* deposits single eggs on isolated and distant plants of various species [27], which increases the risk that *C. glomerata* learns an irrelevant plant odor from a single egg-laying experience into a *P. rapae* caterpillar. For *T. evanescens* the risk of making an irrelevant association is low for both host-species, as the learned cue, an anti-aphrodisiac pheromone, is a highly reliable indicator of future host egg availability.

It is very unlikely that the differences in memory pattern observed are influenced by differences in the conditioned stimuli (CS, in this case odors), as the wasps perceived the CS related to both host species equally well. The composition of the plant odor blend induced by feeding *P. brassicae* and *P. rapae* caterpillars is very similar [34]. It has previously been shown that neither inexperienced *C. glomerata* wasps [35], nor wasps that received a single experience with the plant-host complex [36], distinguish between *P. brassicae*- and *P. rapae*-damaged plants. Furthermore, we found inexperienced *T. evanescens* wasps to be equally attracted to the species-specific odor of mated female *P. rapae* butterflies compared to the odor of mated female *P. brassicae* butterflies (percentage of time spent in the odor field of the mated *P. brassicae* butterflies = 47.8±3.23; WMPSRT, *P* = 0.752). *Trichogramma evanescens* wasps could also learn to prefer the odor of both a *P. rapae* and a *P. brassicae* mated female over the odor of a mated female of the opposite species when tested at 1 h after single-trial conditioning (percentage of time spent in the field of the experienced odor = 57.2±3.51; WMPSRT, *P* = 0.027 when conditioned with *P. rapae*, and 62.0±3.45; WMPSRT, *P* = 0.002 when conditioned with *P. brassicae*), and the preference levels for the learned odors were similar for both host-species (GLM, *P = *0.422).

In most animals for which memory forms have been described so far, long-lasting memory forms (such as ARM and LTM) can consolidate and occur as independent, parallel processes, which together give rise to the observed MR level [12], [37], but see [14]. The neural basis of these different memory forms seems highly conserved [38]. Nevertheless, our previous and current results show that parasitic wasps display a wealth of variation in the dynamics of memory formation. This natural variation offers some unique possibilities to address proximate and ultimate questions in multidisciplinary studies on learning and memory formation [13]. Such an approach, employing natural variation, is a novel contribution to current research on inbred lines, single gene mutants and transgenic animals derived from a few model species. The fact that mechanisms involved in memory formation are highly conserved, ensures that studies on parasitoids are highly relevant [39]. Unlike in most animals, including a closely related parasitoid species, *Cotesia rubecula*, memory transition from ASM to LTM in *C. glomerata* following a single or multiple, spaced egg-laying experiences into *P. brassicae* occurs without an intermediate ARM phase [9], [15]. This suggests that *C. glomerata* consolidates ARM or LTM in a mutually exclusive manner; only ARM after a single experience with *P. rapae* and only LTM after a single or after multiple, spaced experiences with *P. brassicae*. This finding provides excellent opportunities for within-species comparative research into the mechanisms underlying memory consolidation, because exclusive ARM or LTM consolidation processes can be studied after being induced by giving females one single egg-laying experience, either on a *P. brassicae* or on a *P. rapae* caterpillar. Such a comparison constitutes a natural, ecologically relevant approach, but also avoids pleiotropic effects [40], which can compromise the analysis of single-gene mutants and transgenic animals.

We conclude that the memory form that is consolidated does not only change in response to relatively large differences in conditioning, such as the number and type of conditioning trials [10]–[12], but is also sensitive to more subtle differences, such as reward value. Consolidation of specific memory forms should therefore be considered as a plastic trait that, rather than reflecting an animal's capability to learn a specific task, can be adjusted to differences in the perceived value of the reward.

## Materials and Methods

### *Cotesia glomerata* Experiments

#### Insects and Plants

*Cotesia glomerata* wasps (Hymenoptera: Braconidae; approximately 5.0 mm long) were obtained from a colony that originated from individuals collected in cabbage fields in the vicinity of Wageningen, the Netherlands. This laboratory colony, which passes through approximately 15 generations per year, was replaced each year by new field-collected individuals. *Cotesia glomerata* was reared in caterpillars of the large cabbage white butterfly *Pieris brassicae* L. (Lepidoptera: Pieridae). The two butterfly species *Pieris brassicae* and *Pieris rapae* were reared on Brussels sprouts plants (*Brassica oleracea* L. var. *gemmifera* cv. Cyrus, Brassicaceae). Insect rearing was performed in a climatic room at 20–22°C, 50–70% RH and a L16:D8 photoperiod. Upon emergence, male and female parasitoids were caged together to allow mating, and provided with water and honey. Only mated, 3–9 day-old inexperienced female *C. glomerata* wasps were used in the experiments. Brussels sprouts plants and nasturtium plants (*Tropaeolum majus* L. var. Glorious Gleam, Tropaeolaceae) used for the experiments were reared in a greenhouse at 20–25°C, 50–70% RH and a L16:D8 photoperiod.

#### Conditioning Procedure

Conditioning was performed as described previously [41]. A conditioning trial consisted of a single oviposition (egg-laying) experience into a newly emerged *P. brassicae* or *P. rapae* caterpillar that was placed on a damaged leaf of a host feeding-damaged nasturtium plant or a host feeding-damaged Brussels sprouts plant. The Brussels sprouts odor is relatively more attractive to inexperienced wasps than the nasturtium odor [35]. By using two plant species whose odors differ in attractiveness to inexperienced wasps we more closely mimic the natural situation, in which inexperienced wasps always have a strong preference for certain plant odors, and can shift this preference by learning. In our study system, a wasp can learn to increase its preference for nasturtium relative to Brussels sprouts during an oviposition experience with a caterpillar that is feeding on a nasturtium plant. However, a similar experience on a Brussels sprouts plant does not further increase the already existing preference for this plant odor [15]. As inexperienced wasps generally have a low flight response to the plants when tested in the wind tunnel [9], [36], [41], we used wasps conditioned on Brussels sprouts as a reference group. For the 24 h choice test two reference treatments were included, one consisting of wasps conditioned on Brussels sprouts with *P. rapae* and one consisting of wasps conditioned on Brussels sprouts with *P. brassicae*. Because we obtained similar preference levels for these two control treatments, we only included wasps conditioned with *P. brassicae* on Brussels sprouts as a reference group for the 1 h and 4 h choice tests. Two days before their use in the conditioning trials, plants were infested with 40 recently emerged caterpillars that were spread over two leaves. Shortly before conditioning all caterpillars were removed from the plants. Wasps were conditioned with newly emerged caterpillars in order to facilitate oviposition, as older caterpillars tend to defend themselves aggressively. For each conditioning trial a new caterpillar was placed on a damaged leaf. Unconditioned female wasps were individually placed in a glass tube, which was then brought in close proximity to a caterpillar on a damaged leaf. The wasps were released onto the leaf, ensuring direct contact of their antennae with a caterpillar and its products. This stimulation induced an immediate oviposition response, lasting approx. 10 s. Apart from single-trial conditioning, we also included a spaced conditioning treatment, in which *C. glomerata* wasps received three conditioning trials spaced by 10 min intervals. After each oviposition, the parasitized caterpillar was removed and the wasp was transferred to a cage with honey and water until the memory bioassay. Between multiple conditioning trials spaced in time, or when tested 1 h after conditioning, wasps were individually kept in a glass vial.

#### Choice Bioassay

*Cotesia glomerata* wasps were tested at 1 h, 4 h, and 24 h after conditioning in a two-choice wind tunnel set-up [42], in which wasps could fly towards and land on a nasturtium plant or a Brussels sprouts plant, both damaged by host-feeding. Each wasp was only tested once. We tested two six-week old nasturtium plants in a single pot against one eight-week old Brussels sprouts plant, in order to balance differences in size and frontal leaf density. Nasturtium and Brussels sprouts plants were each infested with resp. 20 or 10 newly emerged caterpillars spread over 2 leaves at resp. 2 and 1 day(s) before the choice test. Plants used in the wind tunnel for the 24 h choice test were infested either with *Pieris brassicae* or *P. rapae*, depending on the host species used for conditioning. Plants used for the 1 h and 4 h choice tests, for which only one reference treatment was included, were infested with *Pieris brassicae*, independent of the *Pieris* species used during conditioning. It has previously been shown that *C. glomerata* does not distinguish between *P. brassicae*- and *P. rapae*-damaged plants after a single experience with the plant-host complex [36]. Wasps were released two-by-two into the wind tunnel from the middle of an open glass cylinder that was placed at approximately 70 cm from the two odor sources. After half of the wasps of each conditioning treatment made a choice, plant position was changed in order to prevent bias towards one side of the wind tunnel. Wasps were given maximum 15 minutes to show a preference by flying towards, and landing on, one of the plants. Each experimental day we tested the preference of 10–15 wasps of all conditioning treatments belonging to the same time point or memory-inhibition assay (see below) using the same plant pair (except for the 24 h choice test, in which a different plant pair was used for wasps conditioned with *P. rapae* and for wasps conditioned with *P. brassicae*). For each time point/memory-inhibition assay, wasps were tested on at least 4 different days, resulting in n>40 wasps per conditioning treatment and at least 4 different plant pairs. Memory was defined as present when the percentage of wasps choosing for nasturtium was significantly higher for wasps conditioned on nasturtium compared to wasps conditioned on Brussels sprouts, with a larger difference between these two groups representing a higher memory retention level.

#### Long-Term Memory (LTM)-Inhibition Assay

We used the protein synthesis-inhibitor anisomycin (ANI) to determine whether 4 h memory in *C. glomerata* following single-trial conditioning with *P. rapae* is protein synthesis-dependent. The appropriate ANI concentration had been determined previously [9]. ANI treatment of *C. glomerata* wasps did not affect preference levels of unconditioned wasps, nor did it affect 1 h memory [9]. Female wasps were deprived of honey and water overnight, and fed 0.5 µL of a 3% sucrose solution containing 5 mM ANI the next morning, approximately 2 h before conditioning. Wasps serving as controls for the ANI-fed wasps were given the same treatment, but without ANI in the sucrose solution. Wasps were individually kept in vials for 1 h, and only those wasps that consumed the solution entirely were selected for subsequent conditioning. The selected wasps were transferred to a glass cage with access to water and honey until conditioning. Since 4 h memory in *C. glomerata* following single-trial conditioning with *P. brassicae* is based on LTM [15], we used the wasps conditioned on *P. brassicae* to verify whether the ANI treatment was effective.

#### Anesthesia-Sensitive Memory (ASM)-Inhibition Assay

We used cold-shock induced retrograde amnesia to investigate whether 4 h memory in *C. glomerata* following single-trial conditioning with *P. rapae* is based on ASM or ARM. Cooling wasps on ice for 2 min directly after conditioning disrupts ASM without affecting ARM or LTM consolidation [15]. Such cold-shock induced retrograde amnesia was prominent in *Cotesia* wasps at 20 min after single-trial conditioning [15]. Therefore we compared the wasps' choice distribution at 20 min and 4 h after cold-shock treatment, using different groups of wasps for each time point. Cold-shock applied 10 min before conditioning should not induce amnesia [30] and was used as a positive control for 20 min memory. The nature of our wind tunnel procedure (active oriented flight behavior) ensures that wasps were completely recovered from this treatment at the time of the choice test.

#### Data Analysis

To test for main and interaction effects of “host-species used during conditioning” and “time after conditioning” on memory, we calculated the level of memory retention for each replicate as the difference in the percentage of wasps landing on nasturtium between the group of wasps conditioned on nasturtium and the group of wasps conditioned on Brussels sprouts (the reference group). In this way we could control for the effect of plant pair, as each plant pair differed in relative attractiveness of the nasturtium plant and the cabbage plant, and only wasps belonging to the same time point, but not those belonging to different time points, were tested using the same plant pairs. Memory retention data were analyzed using the generalized linear model (GLM) procedure in SAS v. 8.02 (SAS, Inc., Chicago, IL; http://www.sas.com). A Levene test for homogeneity of variance of treatment groups was carried out, followed by comparisons of least square means of treatment groups that were corrected for type I errors with the Tukey-HSD method. To test whether memory was present at different times after conditioning with *P. rapae* or *P. brassicae* (i.e. whether the percentage choice for nasturtium of the wasps conditioned on nasturtium was higher than that of the reference group) we used a GLM procedure with binomial distribution of error variance and a logit-link function. For each treatment the number of individuals landing on nasturtium in the wind tunnel was used as the response variable, and the number of responding wasps as the binomial total. Data collected on different experimental days (representing different plant pairs) were considered as replicates, and experimental day was introduced as a block factor into the model. In case of overdispersal, the variance functions of the binomial distribution were allowed to have a multiplicative overdispersion factor by dividing the square root of the deviance of the model by the degrees of freedom [43]. Contrast statements were used to separate treatment groups when main effects or their interactions were significant. The same method was used to compare the percentage choice for nasturtium between treatment groups of the memory inhibition assays and between single- and multiple (spaced) conditioning trials. An α = 0.05 level of significance was used for all the comparisons.

### *Trichogramma evanescens* Experiments

#### Insects

*Trichogramma evanescens* Westwood (Hymenoptera: Trichogrammatidae; approximately 0.5 mm long) (iso-female strain GD011) originated from a *P. rapae* egg collected in 2006 in a cabbage field in Wageningen, The Netherlands [23], [24]. Since then, it was reared in eggs of the moth *Ephestia kuehniella* under laboratory conditions (25±1°C, 50–70% rh, L16:D8). With approximately 26 laboratory generations per year and 3–4 years between collection and the execution of the experiments, *T. evanescens* had been in the laboratory for 78–104 generations at the time of the experiments. Only mated, 2-days-old inexperienced female *T. evanescens* wasps were used in the experiments. Mated female and male butterflies of *P. brassicae* and *P. rapae* were taken as copulating pairs the day before a conditioning trial or bioassay. Mated female *Pieris* butterflies are known to emit species-specific anti-aphrodisiac pheromones that are transferred by males during mating to render females less attractive to conspecific males [44].

#### Conditioning Procedure

Wasps were given a conditioning trial that mimics a successful ride on a mated female butterfly as described previously [23]. A trial consisted of first mounting a mated female butterfly, remaining on it during a short simulated flight (the butterfly was relocated with a pair of weak forceps), descending it, and finally laying eggs into one <24 h old butterfly egg of either *P. rapae* (single) or *P. brassicae* (present in a group of eggs) that was deposited on a Brussels sprouts leaf. Wasps carefully assessed egg (cluster) size by walking over – and drumming with their antennae on – one egg (or more eggs in the case of *P. brassicae*) before laying their own eggs. The conditioning procedure took place in a plastic container [23], [24]. Conditioned wasps were individually kept in glass vials with a drop of honey until the memory bioassay.

Apart from single-trial conditioning, we also included a spaced conditioning treatment, in which *T. evanescens* wasps received two conditioning trials spaced by a 1 h interval.

#### Choice Bioassay

We determined memory retention at 1 h, 4 h and 24 h after conditioning in *T. evanescens* by measuring the response of wasps to butterfly odors in an olfactory bioassay that we have developed previously [23], [24]. In a two-chamber olfactometer, two adult mated female butterflies were introduced as odor source in one chamber and two male butterflies in the other. The time spent by the wasps in one of the two odor fields was observed for 300 seconds. Because inexperienced *T. evanescens* do not distinguish between odors of male and mated female butterflies [23], [24], memory was defined as present when the percentage of time spent in the odor field of mated females was significantly higher than 50%, with a larger difference representing a higher memory retention level. We also investigated the possibility that the observed patterns in memory retention are not only related to differences in the reward value, but would also be influenced by differences in the perception of the conditioned stimulus, represented by the species-specific odors of mated female butterflies. We did this by testing the preference of inexperienced wasps, as well as the preference of wasps one hour after they received a single conditioning trial with one of the two butterfly species, in a two-chamber olfactometer with two mated female *P. brassicae* butterflies in one chamber and two mated female *P. rapae* butterflies in the other. Each day 10–15 wasps were tested, until a total of 40 wasps per combination was reached. Each wasp was only tested once. The olfactometer was rotated 180° after every third insect, to compensate for any unforeseen asymmetry in the setup. After each 3^rd^ wasp tested, the butterflies were replaced with new ones.

#### Egg-laying Bioassay

In a no-choice situation, wasps were given the opportunity to parasitize *P. rapae* or *P. brassicae* eggs present on a Brussels sprouts leaf. We determined the number of wasp eggs allocated to a butterfly egg by observing a female wasp's abdominal movements after ovipositor insertion until withdrawal under a microscope as described previously [45]–[47].

#### Data Analysis

To test for main and interaction effects of “host-species used during conditioning” and “time after conditioning” we used a GLM procedure in SAS. The percentage of time that a female wasp spent in the odor field of mated female butterflies was the dependent response variable. Data were arcsine square-root transformed to meet Levene's test for homogeneity of variance of treatment groups. We made Tukey-HSD adjusted pairwise comparisons of least square means of treatment groups. A Wilcoxon's matched-pairs signed-ranks test (WMPSRT) was used to test whether the percentage of time spent in the odor field of mated female butterflies was significantly higher than 50%. To compare the number of eggs allocated to a *P. brassicae* with those allocated to a *P. rapae* egg we used a non-parametric Mann-Whitney U test for non-related samples. An α = 0.05 level of significance was used for all the comparisons.

## Supporting Information

Movie S1 **Oviposition behavior of female** ***Cotesia glomerata*** **wasps into first instar caterpillars of** ***Pieris brassicae*** **.** These caterpillars of the large cabbage white butterfly *P. brassicae* are feeding gregariously on a leaf of Brussels sprouts, and are attacked by two *C. glomerata* females. The very fast insertion of the ovipositor into the caterpillars is clearly visible. The oviposition experience serves as a reward in associative learning, where plant odors are learned to predict the presence of suitable hosts [9], [15]. The parasitic wasp lays approximately 20 eggs in each caterpillar, after which the caterpillar will continue to develop until the fifth larval instar. When the parasitic wasp larvae are fully-grown they will emerge from the caterpillar, spin a cocoon and develop into adult parasitic wasps. The caterpillar will die before pupation. Movie (41 seconds) courtesy of Hans M. Smid.
(MOV)

Movie S2 **Oviposition behavior of a female** ***Trichogramma*** **wasp into a** ***Pieris brassicae*** **egg.** Eggs of the large cabbage white butterfly *P. brassicae* are normally present within a clutch of 20–50 eggs. For a better view the butterfly egg was isolated from the clutch. After the first contact a female wasp walks over - and drums with her antennae on - the butterfly egg to assess its size [32]. She then carefully adjusts the number and sex of the eggs that she oviposits into the butterfly egg accordingly [45]–[48]. After oviposition, the wasp eggs will develop into adult wasps inside the butterfly eggs at the cost of caterpillar development. Movie (41 seconds) courtesy of Nina E. Fatouros, Martinus E. Huigens and Urs Wyss (Institute of Phytopathology, University of Kiel, Germany).
(MOV)

Movie S3 **Mounting behavior of female** ***Trichogramma*** **wasp on a mated** ***Pieris brassicae*** **female.** *Trichogramma* wasp mounts a mated female of the large cabbage white butterfly *Pieris brassicae* to hitch a ride to the butterfly's oviposition sites where they parasitize the freshly laid eggs [22]–[24]. In case wasps try to climb onto the butterfly's leg, they can be kicked off. Therefore wasps mostly climb onto the butterfly's wings after which they tend to move towards the thorax just behind the butterfly's head. Movie (50 seconds) courtesy of Nina E. Fatouros, Martinus E. Huigens and Urs Wyss (Institute of Phytopathology, University of Kiel, Germany).
(MOV)
